# Tumor and Endothelial Cell-Derived Microvesicles Carry Distinct CEACAMs and Influence T-Cell Behavior

**DOI:** 10.1371/journal.pone.0074654

**Published:** 2013-09-11

**Authors:** Harrison T. Muturi, Janine D. Dreesen, Elena Nilewski, Holger Jastrow, Bernd Giebel, Suleyman Ergun, Bernhard B. Singer

**Affiliations:** 1 Institute of Anatomy, University Hospital Essen, Essen, Germany; 2 Institute for Transfusion Medicine, University Hospital Essen, Essen, Germany; 3 Institute of Anatomy and Cell Biology, Julius-Maximilians-University Würzburg, Würzburg, Germany; Ludwig-Maximilians University, Germany

## Abstract

Normal and malignant cells release a variety of different vesicles into their extracellular environment. The most prominent vesicles are the microvesicles (MVs, 100-1 000 nm in diameter), which are shed of the plasma membrane, and the exosomes (70-120 nm in diameter), derivates of the endosomal system. MVs have been associated with intercellular communication processes and transport numerous proteins, lipids and RNAs. As essential component of immune-escape mechanisms tumor-derived MVs suppress immune responses. Additionally, tumor-derived MVs have been found to promote metastasis, tumor-stroma interactions and angiogenesis. Since members of the carcinoembryonic antigen related cell adhesion molecule (CEACAM)-family have been associated with similar processes, we studied the distribution and function of CEACAMs in MV fractions of different human epithelial tumor cells and of human and murine endothelial cells. Here we demonstrate that in association to their cell surface phenotype, MVs released from different human epithelial tumor cells contain CEACAM1, CEACAM5 and CEACAM6, while human and murine endothelial cells were positive for CEACAM1 only. Furthermore, MVs derived from CEACAM1 transfected CHO cells carried CEACAM1. In terms of their secretion kinetics, we show that MVs are permanently released in low doses, which are extensively increased upon cellular starvation stress. Although CEACAM1 did not transmit signals into MVs it served as ligand for CEACAM expressing cell types. We gained evidence that CEACAM1-positive MVs significantly increase the CD3 and CD3/CD28-induced T-cell proliferation. All together, our data demonstrate that MV-bound forms of CEACAMs play important roles in intercellular communication processes, which can modulate immune response, tumor progression, metastasis and angiogenesis.

## Introduction

A broad range of cell types including epithelial and endothelial cells, leukocytes and tumor cells are able to release at least three major types of extracellular vesicles. Vesicles derived from the endosomal system are termed exosomes and have a diameter of 70-120 nm [1,2]. Per definition exosomes originate from late endosomes, which upon their maturation bud small vesicles, the intraluminal vesicles, into their interior. Accordingly such endosomes are also termed multivesicular bodies (MVBs). Upon fusion of the outer membranes of the MVB with the plasma membrane they can release their intraluminal vesicles as exosomes into their environment [3]. Exosomes can be released constitutively or upon induction [4].

With 100-1 000 nm in diameter microvesicles (MVs) are larger in size than exosomes [4]. MVs are shed from the cell membrane. MV shedding is a physiological phenomenon that accompanies cell activation and growth. Their secretion can be increased by stress factors such as cell activation, hypoxia, lack of nutrition, irradiation, oxidative injury, and subsequent increase of cytosolic Ca^2+^ [5,6]. Released microvesicles have been isolated and characterized from cultured cell lines as well as from various body fluids including blood plasma, serum, urine, amniotic fluid, bronchoalveolar fluid, and tumor effusion [4,5]. Increased levels of MVs have been detected in peripheral blood of patients suffering from tumors with highly metastatic potential [7–9]. A third class of cell-derived microvesicles is the apoptotic bodies, which are released as blebs of cells undergoing the programmed cell death. In contrast to the other types of vesicles apoptotic bodies are considerably larger at ~ 1-5 µm in diameter and contain DNA fragments and organelles, like mitochondria, lysosomes and ribosomes [10–12].

In this study we focused on analyzing MVs. MVs play an important role in modulating numerous cellular processes, such as angiogenesis, tumor progression and metastasis, cancer immune suppression, tumor-stroma interactions, and further biological processes [13]. Analogous physiological and pathological functions have been shown for members of the carcinoembryonic antigen (CEA)-related cell adhesion molecule (CEACAM) family.

CEACAMs belong to the immunoglobulin (Ig) superfamily and thus appear as highly glycosylated proteins with the typical N-terminal variable Ig-like domain followed by 0 to 6 constant Ig-like domains [14,15]. A hydrophobic transmembrane domain with a cytoplasmic tail (CEACAM1-CEACAM4) or a glycosylphosphatidylinositol (GPI) lipid moiety (CEACAM5-CEACAM8) anchors CEACAMs to the cell membrane [14,16,17]. The transmembrane bound CEACAMs can mediate signal transduction utilizing their cytoplasmic phospho-tyrosine based signaling motifs (ITIM in CEACAM1, ITAM in CEACAM3) [18–21]. CEACAMs function as low affinity homophilic and heterophilic cell-cell adhesion receptors that often act as co-receptors e.g. of the T-cell receptor [22], B-cell receptor [23], TLR-2 [24], TLR4 [25], VEGFR1 [26,27], VEGFR2 [28], VEGFR3 [29], EGFR [30], insulin receptor [31,32] and the GM-CSFR [33]. CEACAMs can be found in epithelia, angiogenically activated endothelia, and most leukocyte subtypes [20,34,35], although the CEACAM expression pattern varies significantly between these cell types. In human, epithelia express CEACAM1, CEACAM5, CEACAM6 and CEACAM7, while granulocytes express CEACAM1, CEACAM3, CEACAM6 and CEACAM8. In contrast, lymphocytes and activated endothelial cells only express CEACAM1 [16,26,36,37]. The CEACAM expression in other species is mostly restricted to CEACAM1 [36]. Therefore CEACAM1 is regarded as the ancestral founder molecule of the CEACAM family [36].

So far no data exist on the presence and functions of CEACAMs on MVs. Therefore we investigated the CEACAM expression pattern on tumor and endothelial cell-derived MVs of different species. Furthermore we analyzed the signaling properties of CEACAM1 in MVs and the function of tumor derived CEACAM positive MVs on T-lymphocytes. We found that CEACAM1, CEACAM5 and CEACAM6 are present on tumor-derived MVs, and that human and murine endothelial cells release CEACAM1 expressing MVs. Pervanadate treatment of MVs did not provoke tyrosine phosphorylation of CEACAM1 suggesting a lack of its signal transduction capability. However, CEACAM-positive MVs increased the proliferative response of T-cells stimulated by CD3/CD28.

## Results

### Morphological examination of MVs

**Figure 1:**
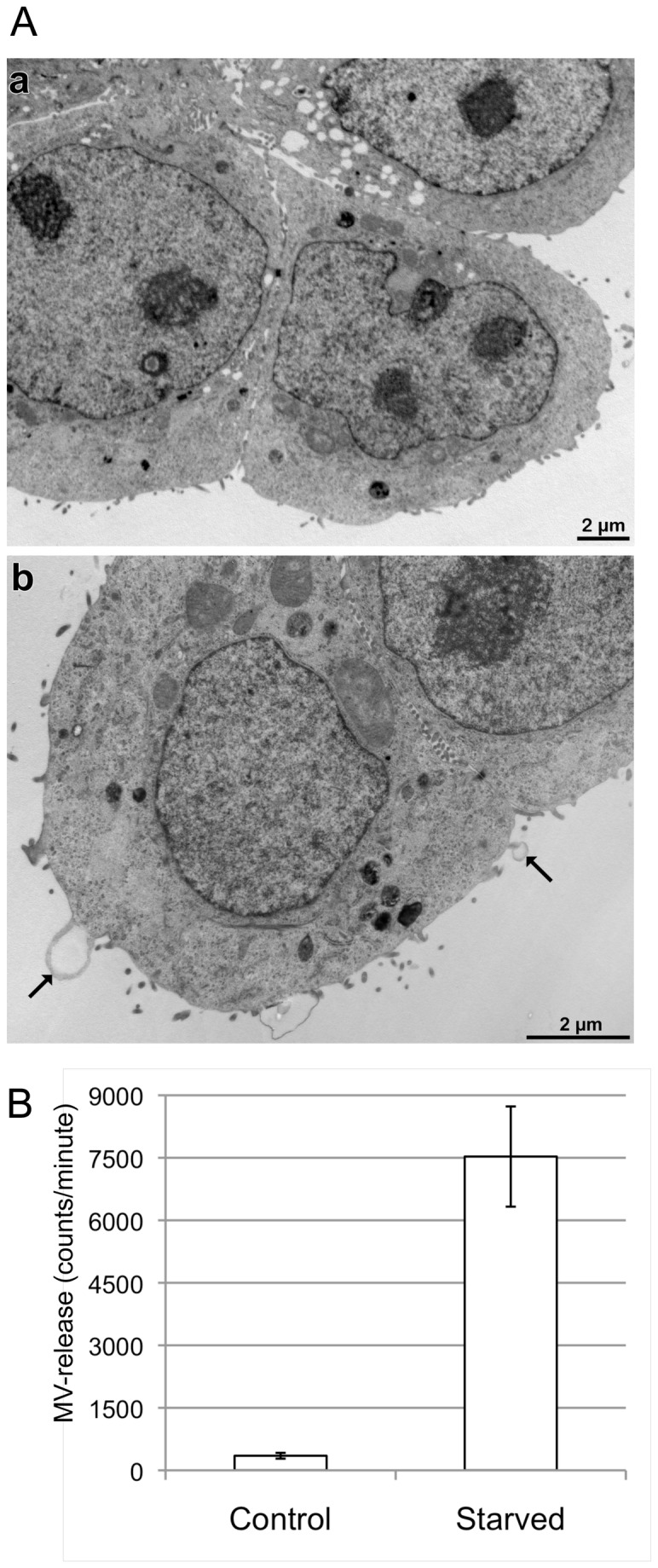
Colon tumor epithelial cells release MVs in response to serum starvation. A) Transmission electron micrographs of HT29 cells grown under normal conditions (a) and serum starved (b) for 48 h. After culture, cells fixed, embedded in mold, cut in thin sections and analyzed by transmission electron microscopy. B) The amount of MVs released by HT29 cells cultured in media with or without serum was counted by flow cytometry (n=3).

MVs are released from luminal cell surface membranes when protrusions bleb off. In order to observe this process, HT29 cells were cultured on a Thermanox coverslip for 3 days, and prepared for electron microscopy, as described in Materials and Methods. Transmission electron microscopy showed chubby protrusions of luminal cell membranes with electro-lucent content and little texture indicating a high percentage of water. Eventually, a few spherical vesicles with similar light content were present in the media, i.e. MVs. Our results showed no significant budding off of MVs in control cells cultured with FCS containing medium (Figure 1A, panel a). Cells cultured in FCS free medium revealed circular structures located closely to, or protruding from, the outer cell membrane (Figure 1A, arrows panel b). Note the two distinct sites where protrusions with mainly electron lucent, i.e. water-rich, content appear. These are a direct continuation of the much more electron-dense cytoplasm that kind of surrounds the watery region (Figure 1A panel b, arrows). We concluded that these circular structures are MVs released from the cell membrane. Following this idea we collected the same volume of cell culture supernatants of starved and non-starved HT29 cells and determined the amount of MVs counted by a flow cytometer within one minute (counts per minute, c/m). As shown in Figure 1B control cells showed barely any MVs in the supernatant (350 ± 70 c/m, n=3) whereas starved cells release significant amounts of MVs (7530 ± 1200 c/m, n = 3). These results clearly show that starving cells induced the release of MVs.

**Figure 2:**
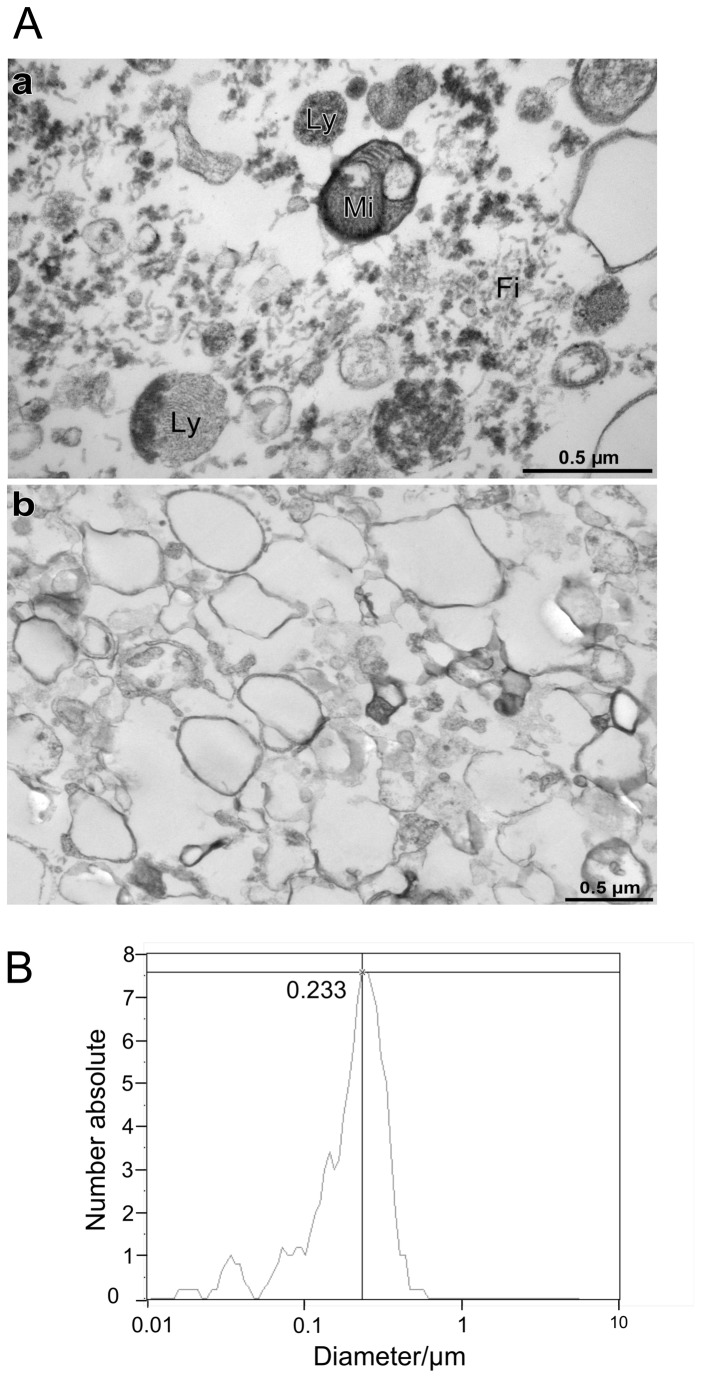
Introduction of a filtration step into the MVs isolation protocol significantly improves pureness of the isolates. A) Transmission electron micrographs of MVs isolated from serum starved HT29 supernatants by centrifugation without (a) or with (b) prior 0.8 µm filtration step. Samples were fixed, embedded in mold, cut in thin sections and observed using transmission electron microscopy. B) MVs filtered and isolated from serum starved HT29 cells were analyzed with the nanoparticle tracking analysis, allowing estimating the average size distribution of MVs.

Next we developed a method to isolate MVs without potential contamination with apoptotic bodies. The common procedure for the isolation of MVs from cell culture supernatants involves differential centrifugation [38]. Unfortunately probes isolated by this technique are known to be contaminated by microsomal fractions and organelles released from apoptotic cells. To avoid such contaminations in the MVs isolates, we introduced a special filtration step into our MVs isolation method. To show the benefit of this filtration step we collected the supernatant of HT29 cells cultured for 3 days in starvation medium. After centrifugation at 2860 g for 20 minutes at 4°C the sample was divided in two fractions. One supernatant fraction was filtered through a 0.8 µm sterile filter whereas the other was left untreated. Thereafter both supernatant fractions were centrifuged at 41 000 g for 1 hour at 4°C. Then the pellet was prepared for electron microscopy. Analyzes of the ultrathin sections demonstrated that MVs harvested from the fraction without filtration were contaminated with considerable amounts of cytoplasmic residues like mitochondria (Mi), heterolysosomes (Ly) and high amounts of most likely actin filaments (Fi) (Figure 2A panel a). In contrast, MVs isolated from the filtered supernatant did not show significant amounts of organelles or other contaminations (Figure 2A panel b). Thus, our data clearly show that the introduction of a filtration step before centrifugation is important for contaminant free MVs isolation.

Next we utilized the nanoparticle tracking analysis (ZetaView PMX 100 system, Microtrac Europe GmbH) to determine the size of the isolated MVs. Our data showed an average mean diameter of 233 nm for more than 90% of all MVs isolated from supernatants of starved HT29 cells (Figure 2B). Only 5% of the MVs had less than 100 nm in average mean diameter and 3% more than 800 nm following our MVs isolation protocol. These data reflect the purity and homogeneity of our isolates seen by electron microscopy. In general, a size range of MVs from 100 nm to 1 000 nm has been reported [4].

To verify the sizes of isolated MVs shown above we performed electron microscopy of three different HT29 MVs isolates and measured the diameters of 130 MVs. Here we found a very heterogeneous mixture, with diameters ranging from 90 nm to 812 nm, with an average of 320 nm ± 164 nm (n = 130).

### Epithelia, endothelia and CHO-CEACAM1 derived MVs express distinct CEACAM expression patterns

**Figure 3:**
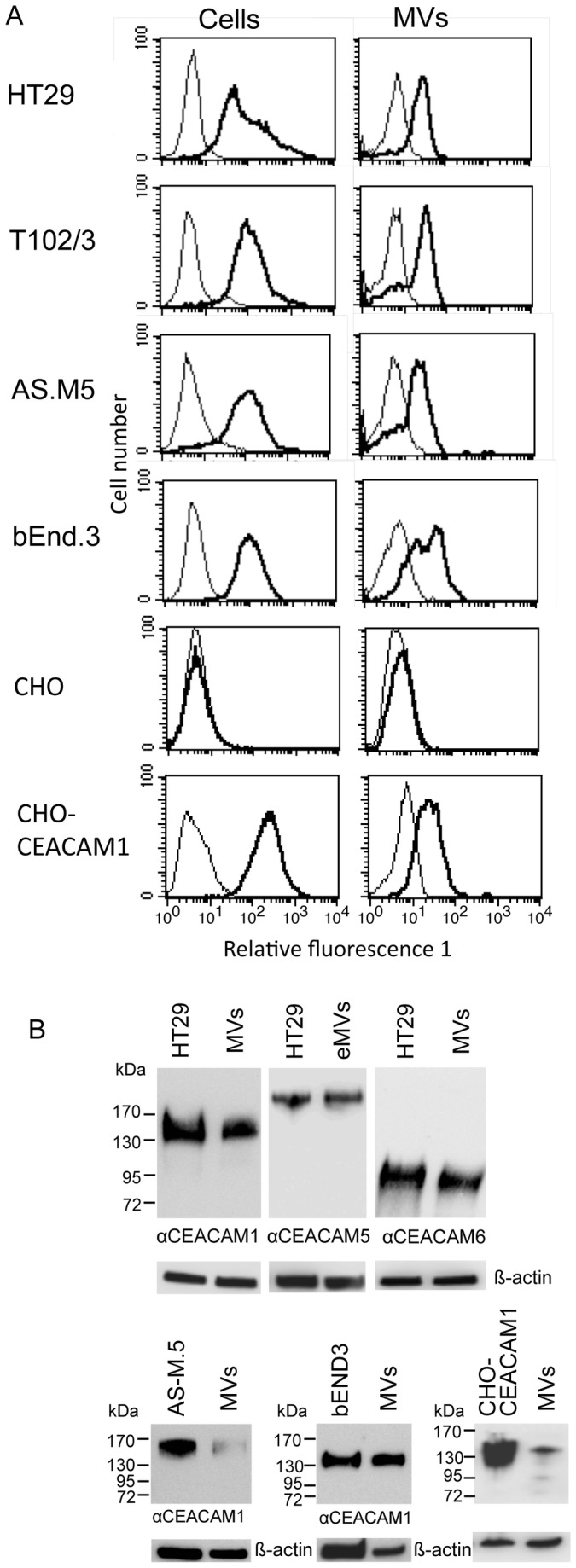
MVs released by CEACAM expressing cell types reveal a parental cell like CEACAM expression pattern. Human epithelial cell (HT29, T102/3), human endothelial cells (AS-M.5), mouse endothelial cells (bEND3), CHO and CHO-CEACAM1 cells were serum starved for 48 h. Subsequently culture supernatants were filtered (0.8 µm) before MVs were isolation. Harvested cells and MVs were analyzed by flow cytometry (A). Cells were stained for CEACAM1 (thick line). Background fluorescence was determined by incubating the cells with control IgG antibody instead of anti-CEACAM1 antibody (thin line). (B) Parts of obtained samples were lysed and then analyzed by Western blot using anti-CEACAM1-, CEACAM5- and CEACAM6- specific mAbs. Beta-actin detection served as control for equally loading. The experiments shown are representative for three independent repeats.

Similar biological functions have been implicated for MVs and members of the CEACAM family [14,34,39]. Therefore we examined whether CEACAMs are present on MVs of epithelial and endothelial origin. In addition, we analyzed both human and murine endothelial cell lines to see if the assumed CEACAM expression on MVs appears similarly in different species. To examine if CEACAM1 positive MVs can be generated by transfection, MVs released by CHO- and CHO-humanCEACAM1-4L were tested for the presence of CEACAM1. Utilizing flow cytometry, we first analyzed the surface expression of CEACAM1 in the different cell types and MVs derived thereof. Our results showed significant expression of CEACAM1 in the colon tumor cell lines HT29 and T102/3, the human endothelial cell line AS-M.5, the mouse endothelial cell line bEnd.3 and the CHO-huCEACAM1-4L cell line with an expression level of 64, 81, 87, 91 and 164 (median value of the relative fluorescence), respectively (Figure 3A). MVs generated of HT29, T102/3, AS-M.5, bEnd.3 and CHO-CEACAM1-4L cells revealed a CEACAM1 expression level of 23, 26, 18, 25 and 21 (median value of the relative fluorescence), respectively (Figure 3A). As expected, CHO cells and their MVs were negative for human CEACAMs. The isotype matched control IgG for all cell and MVs samples was set to a median value of the relative fluorescence of 4. These results were confirmed by CEACAM1 specific Sandwich-ELISA (data not shown). Furthermore, we found significant expression of CD49b (integrin alpha 2) and CD29 (integrin beta 1) on HT29 cells and MVs released by HT29 cells (data not shown). CD49b and CD29 are both proteins known to be present on MVs and thus served as positive control [40]. Because it was known that HT29 express CEACAM1, CEACAM5 and CEACAM6 we performed Western blot analysis on lysates of HT29 cells and HT29 derived MVs. The samples were probed with antibodies monospecific for CEACAM1, CEACAM5 and CEACAM6. Beta actin detection served as control for equal loading (Figure 3B). Both HT29 cells, and MVs derived thereof, expressed CEACAM1, CEACAM5 and CEACAM6 (Figure 3B, upper panel). Lysates of T102/3 cells and MVs generated thereof also stained positive for CEACAM1, CEACAM5 and CEACAM6 (data not shown). In both cases the molecular weight of the respective CEACAM found in MVs was identical to the molecular weight found in the parental tumor cell line. Thus, our data show that tumor cell derived MVs carry the same expression pattern of CEACAMs on their surface as the cell type they were derived from. Analyses of CEACAM1 in lysates of the human endothelial cell line AS-M.5 and their MVs displayed the same molecular weight of the protein (Figure 3B, lower left panel). The same was true for the murine endothelial cell line bEND3, demonstrating that the release of CEACAM positive MVs was not restricted to the human system (Figure 3B, lower middle panel). CHO-CEACAM1-4L and their MVs also revealed a significant CEACAM1 expression (Figure 3B, lower right panel). Taken together, our results clearly show the presence of CEACAMs on MVs. Furthermore, the CEACAM expression pattern in MVs reflected the one found on the parental cells of which they were derived from.

### Pervanadate and peroxide do not induce tyrosine phosphorylation in MVs, but MVs induce CEACAM1 tyrosine phosphorylation in epithelial cells

**Figure 4:**
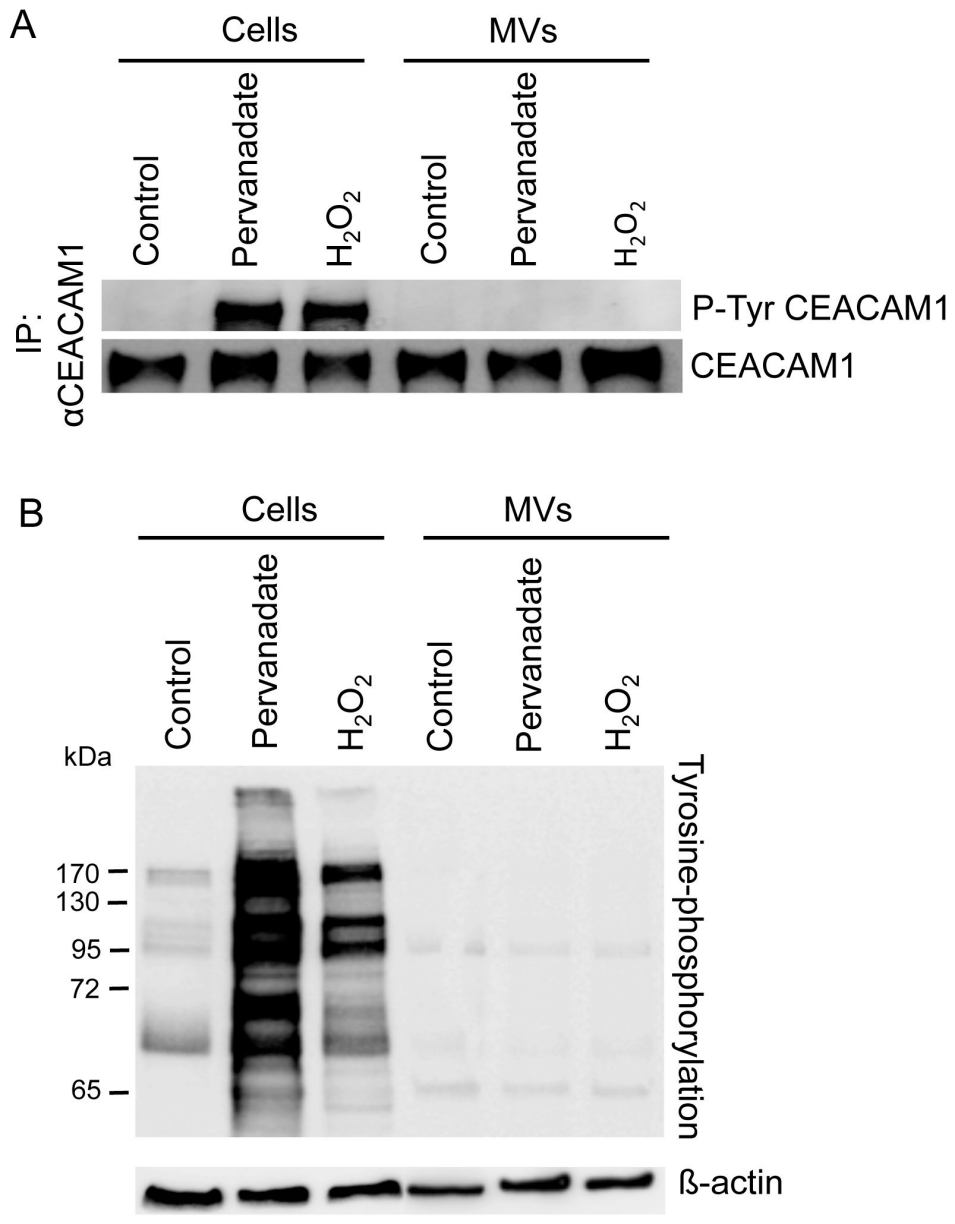
Pervanadate and peroxide treatment induce tyrosine phosphorylation in cells but not in corresponding MVs. Confluent HT29 cells and corresponding MVs were treated with pervanadate or H_2_O_2_, respectively, or left untreated. Western blot analyzes were performed using mAb 4G10 for detecting tyrosine phosphorylation in A) the CEACAM1 immunoprecipitates, and B) the whole cell and MVs lysates. In A) CEACAM1 detection and in B) beta-actin detection served as loading control. The data show one representative result of three independent repeats of the experiment.

**Figure 5:**
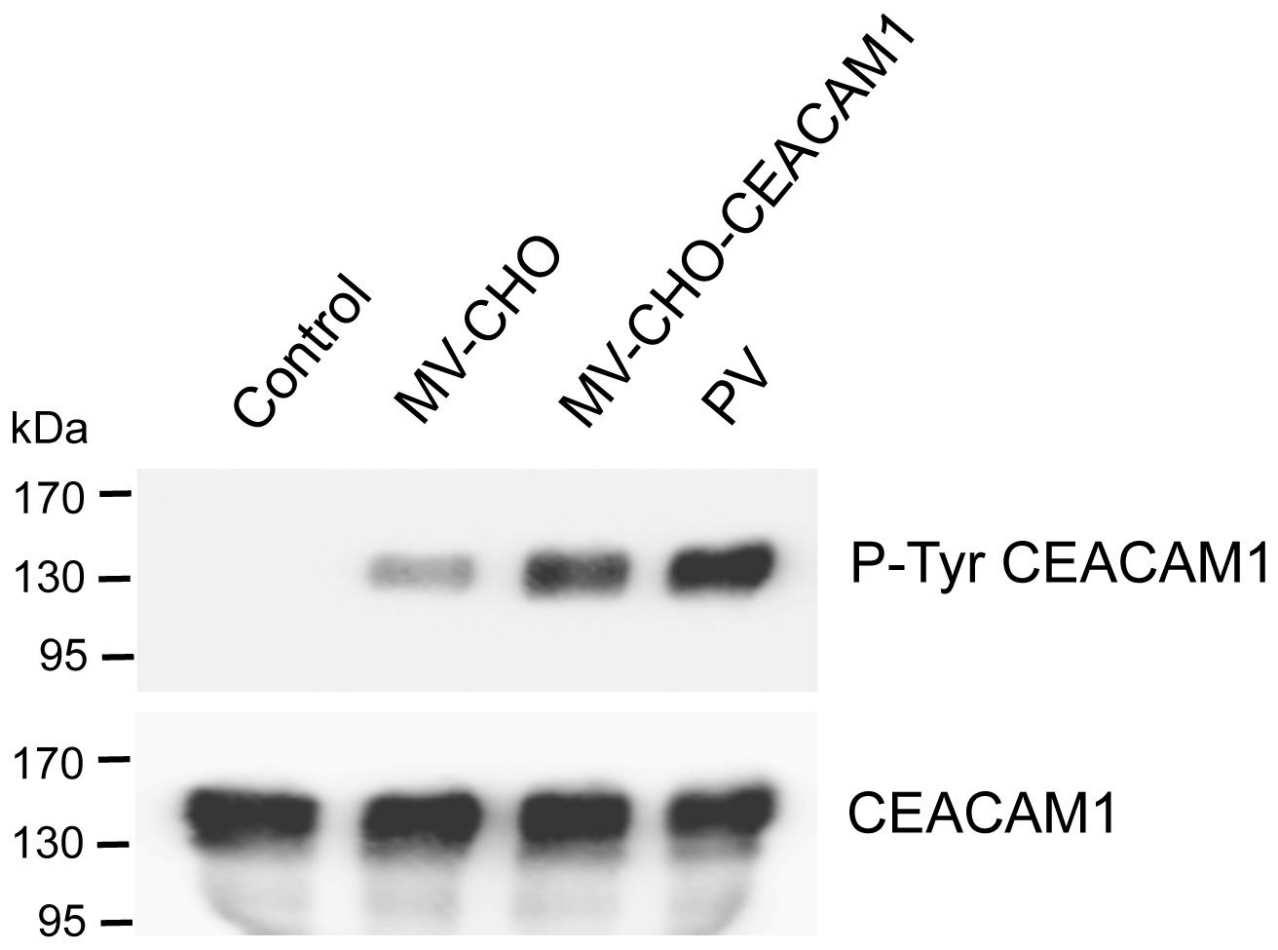
CHO- and CHO-CEACAM1 derived MVs induce the CEACAM1-L tyrosine phosphorylation in confluent HT29 cells. CEACAM1 immunoprecipitates of confluent HT29 cells treated for 15 min with CHO- and CHO-CEACAM1 derived MVs were probed for tyrosine phosphorylation (upper panel) and CEACAM1 (lower panel) as control for equal loading. Untreated cells were used as negative control. Pervanadate was used to induce CEACAM1-L tyrosine phosphorylation. The data show one representative result of three independent repeats of the experiment.

Expressed in cells, CEACAM1-L can be phosphorylated on its Tyr residues in response to various stimuli [41]. Association of CEACAM1-L with protein-tyrosine kinases of the Src family and the protein-tyrosine phosphatases SH2-containing Tyr phosphatase-1 (SHP-1) and SHP-2 [19,42] were described as initial steps of the CEACAM1 triggered signal transduction [24]. To analyze whether CEACAM1 in MVs becomes tyrosine phosphorylated we treated isolated HT29 derived MVs with pervanadate and H_2_O_2_, respectively. As positive control the same treatment was performed on confluent HT29 cells. Untreated HT29 cells and HT29 derived MVs served as negative controls. The samples were analyzed for tyrosine phosphorylated CEACAM1 performing an immunoprecipitation followed by immunoblotting. As expected, in HT29 cells tyrosine phosphorylation of CEACAM1-L was induced by pervanadate and H_2_O_2_ treatment but not in untreated probes (Figure 4A, left panel). In contrast, no tyrosine phosphorylation of CEACAM1 was found in pervanadate or H_2_O_2_ treated and untreated MVs (Figure 4A, right panel). Determining the amount of CEACAM1 in the samples verified equal loading (Figure 4A, lower panel). To analyze if any pervanadate and H_2_O_2_ induced tyrosine phosphorylation appears in MVs, we analyzed the lysates of the different samples by immunoblotting. As shown in Figure 4B, a clear pattern of tyrosine phosphorylation was found in lysates of un-stimulated HT29 cells. However, the basal level of tyrosine phosphorylation was clearly increased upon stimulation; pervanadate triggered a stronger effect than H_2_O_2_. Although a pattern of weak tyrosine phosphorylated proteins appeared in HT29 derived MVs, no tyrosine phosphorylation was induced by pervanadate and H_2_O_2_ (Figure 4B upper panel). Equal loading was confirmed by measurement of beta-actin (Figure 4B lower panel). The results shown here were further confirmed by analyzing AS-M.5 cells and AS-M.5 derived MVs showing a similar outcome (data not shown). Thus, neither CEACAM1 nor other proteins present in MVs become tyrosine phoshorylated in response to stimulation. Of note, treatment with both, CHO- and CHO-CEACAM1 derived MVs induces a significant tyrosine phosphorylation in CEACAM1-L expressed on confluent HT29 cells (Figure 5). Since the effect was much stronger with CEACAM1-positive MVs we concluded that in association with homophilic CEACAM1-CEACAM1 interactions, CEACAM1 independent mechanisms can induce CEACAM1-L tyrosine phosphorylation. Thus, CEACAM1-L itself is not tyrosine phosphorylated in MVs but as a homophilic ligand on MVs it augments tyrosine phosphorylation of cellular CEACAM1-L.

### The presence of tumor derived MVs and CEACAM1-positive MVs in anti-CD3/CD28 mAb stimulated PBMCs leads to increased cell proliferation

**Figure 6:**
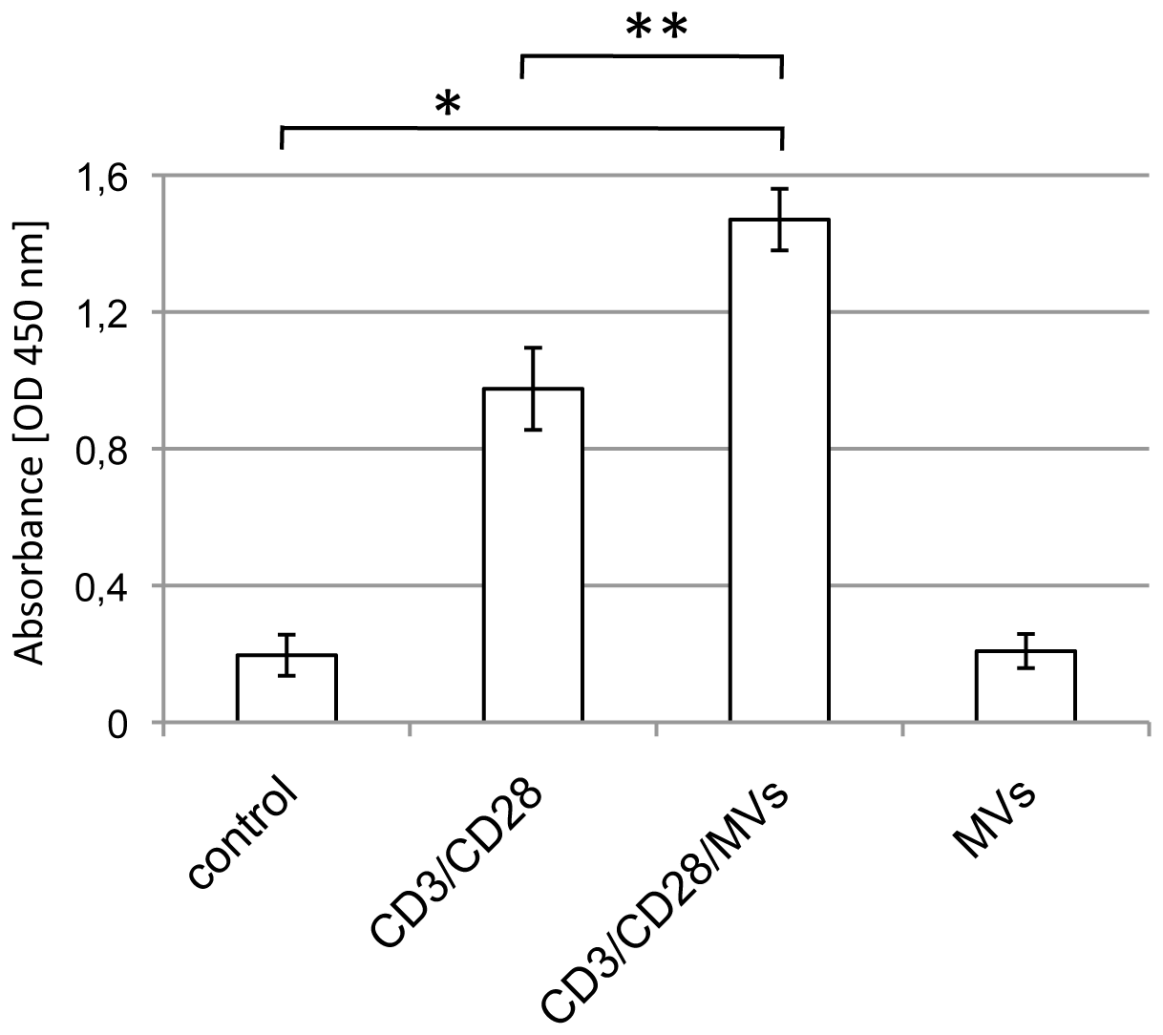
Tumor cell derived MVs promote the proliferative impact of anti-CD3/CD28 mAb stimulation on PBMC. Freshly isolated human PBMC were kept for 3 days in the presence of CD3/CD28, CD3/CD28/MVs and MVs alone. As control untreated cells were used. For the last 14-18 h of cell culture the BrdU compound (Roche) was added. Then the cell proliferation assay was performed according to the manufacturer’s protocol. Experiments were performed in triplicates and results presented are expressed as means of OD 450 nm ± SD. The data show one representative result of four independent repeats of the experiment. The difference between the control group and the CD3/CD28 and CD3/CD28/MVs treated groups as well as the CD3/CD28 and CD3/CD28/MVs treated groups were determined by student t-test (*p≤0.005 and **p≤0.0065, respectively; n=3).

**Figure 7:**
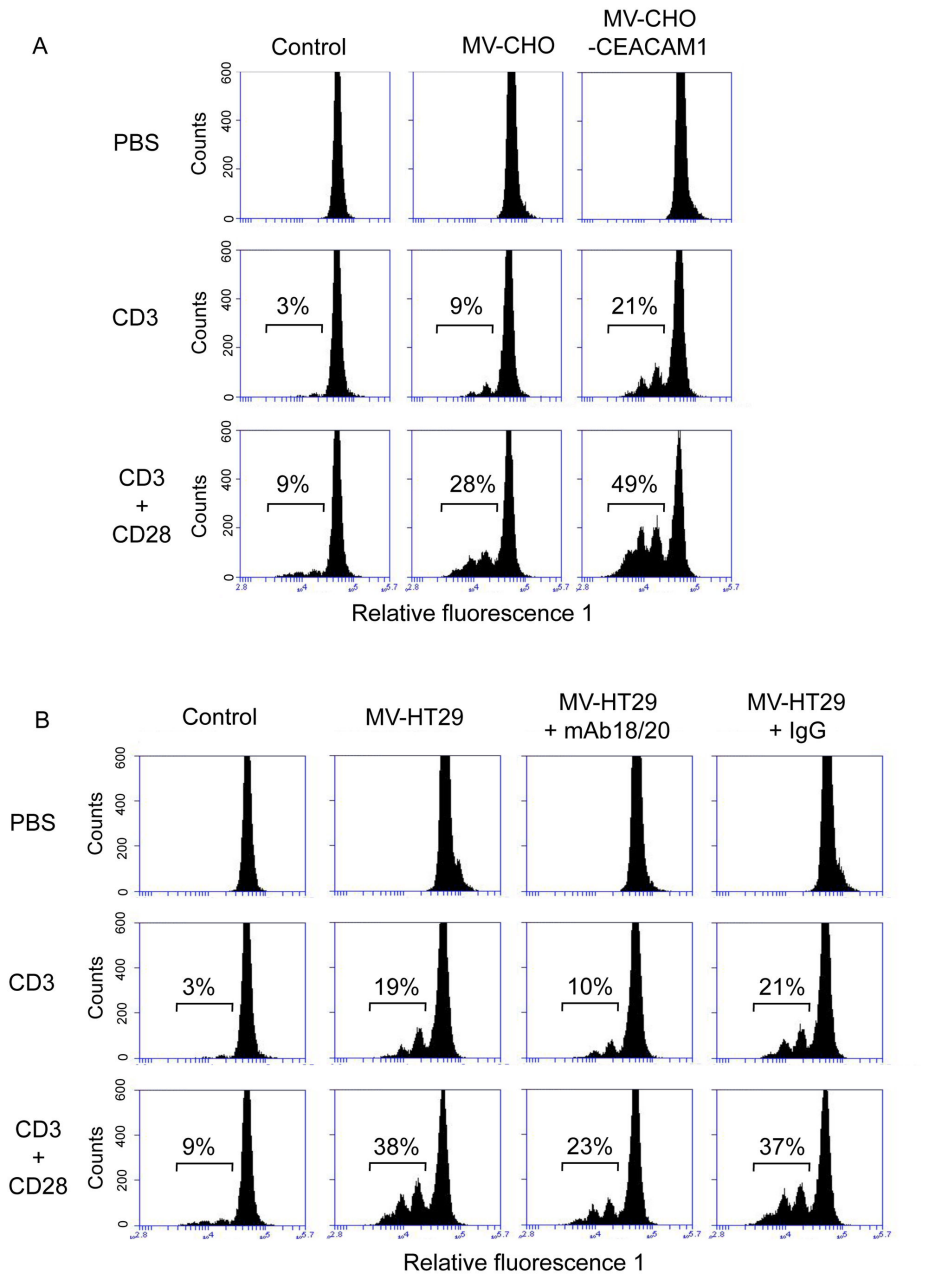
CEACAM1-positive MVs significantly increase the anti-CD3 and anti-CD3/CD28 mAb triggered T-cell proliferation. Freshly isolated human PBMC were labeled with CFSE and cultured for 4 days in the presence of anti CD3 and anti CD3/CD28 with and without CHO- and CHO-CEACAM1 derived MVs (A). B) CFSE labeled PBMC were cultured for 4 days in the presence and absence of antiCD3 and antiCD3/CD28 with and without HT29-derived MVs. Untreated treated cells served as control. In indicated cases samples were co-cultured with antiCEACAM1 mAb18/20 (50 µg/ml) or isotype matched control IgG (50 µg/ml). Then PBMCs were analyzed utilizing the Accuri C6 flow cytometer system. The histograms depict PBMCs that have divided 1-3 times based on CFSE dilution peaks and reflex the cell proliferation rate given in %. The data shown are representative for three independent repeats of the experiment.

To get first insights into the functional role of our highly pure human epithelial tumor cell derived MVs we performed proliferation tests utilizing the anti-CD3 or anti-CD3/CD28 mAb driven PBMC stimulation approach. Treatment of freshly isolated PBMCs with HT29 or CHO/CHO-CEACAM1 derived MVs for three days showed no mitogenic effect in a BrdU or a CFSE assay, respectively (Figures 6 and 7A) and did not alter the cell survival rate (data not shown). In contrast, anti-CD3 and anti-CD3/CD28 antibody treatment induced PBMC proliferation depending on the amount of the seeded cells relatively strong (Figure 6) or only marginally (Figure 7A). Co-treatment of anti-CD3 and anti-CD3/CD28 mAb with HT29 or CHO/CHO-CEACAM1 derived MVs (Figures 6, 7A, 7B) significantly increased induced cell proliferation. Remarkably, the proliferation promoting impact of CHO-CEACAM1 MVs was clearly higher than that of CHO MVs (Figure 7A). It should be noted that PBMCs of a few donors did show a non-significant inhibition of the CD3/CD28 triggered proliferation (data not shown). Next, we analyzed if the T-cell co-stimulatory effect of CEACAM1-positive, HT29 derived MVs could be prevented by the human CEACAM1 binding mAb18/20. The presence of mAb18/20 and the isotype-matched control IgG did not alter the PBMC proliferation rate (not shown). Anti-CD3 antibody alone or in combination with anti-CD28 antibodies showed low induction of PBMC proliferation (Figure 7B, 3% and 9%, respectively). The presence of HT29-derived MV significantly increased the CD3 and CD3/CD28 triggered proliferation rate to 19% and 38%, respectively (Figure 7B). This co-stimulatory effect was reduced by mAb18/20 treatment to 10% and 23%, respectively. In contrast, the control IgG did not change the co-stimulatory effect of HT29-MVs (21% and 37%, respectively). Thus, the CD3-costimulatory effect of tumor derived MVs was at least partially inhibited by CEACAM1 binding antibodies (Figure 7B).

## Discussion

In this study we demonstrate for the first time the presence of CEACAM family members on MVs released by epithelial and endothelial cells of human and mouse origin. MVs are shed from almost all cell types including tumor cells by budding off the luminal cell membrane. They appear as spherical extracellular nanovesicles bordered by a phospholipid membrane. Multiple biochemicals as well as cellular stress, like lack of nutrition and hypoxia, induce an increased release of MVs [4]. Because MVs normally reflect the antigenic content of the cells from which they originate, members of the CEACAM family expressed in epithelia, endothelia, and hematopoietic cells should be present. Since the human colon epithelial cell line HT29 was known to express CEACAM1, CEACAM5 and CEACAM6 we analyzed the MVs generation and the CEACAM expression pattern in HT29 derived MVs. Here we show electron microscopic proof that HT29 release MVs. Serum starvation of HT29 cells triggered a massive shedding of MVs. Similar results were found for the human colon epithelial cancer cell line T102/3, as well as the human endothelial and murine endothelial cell lines AS-M.5 and bEND3, respectively. These findings are in accordance with recent publications describing the constitutive and stimulation-increased release of MVs [5]. Interestingly, blood from cancer patients also contains elevated levels of cell-derived MVs, making our cell culture system physiologically relevant [5]. Investigation by flow cytometry, Western blot and ELISA revealed expression of CEACAM1, CEACAM5 and CEACAM6 in HT29 and T102/3 derived MVs, as well as CEACAM1 expression in endothelial cell derived MVs. Although evidence for the presence of CEACAM5 in tumor derived MVs has been previously reported, no thorough analysis on CEACAMs in MVs has been published [43]. The CEACAM expression in MVs was not restricted to humans but was also found in mice. Accordingly, the CEACAM expression profile in MVs reflected the expression pattern of the cell type they were derived from.

Our studies were performed on MVs isolated from 0.8 µm filtered cell supernatants centrifuged for 1 hour at 41 000 g and 4°C. In the beginning we followed standard isolation protocols found in the literature utilizing centrifugation conditions from 18 000 g to 100 000 g for one hour [38]. Unfortunately, MVs isolated following such protocols resulted in isolates containing many contaminants e.g. with mitochondria and heterolysosomes. Introducing a 0.8 µm filtration step into our MVs isolation procedure greatly improved the purity of the isolates allowing us to produce MVs populations with barely any contamination. The size determinations utilizing the novel Microtrac ZetaView PMX 100 system showed that over 90% of the isolated MVs had an average diameter of 233 nm. Therefore we concluded that HT29 derived MVs were fairly homogeneous in size. Although the electron microscopic MVs size determination resulted in an average diameter of 320 nm, a value, which was near the one measured with the Microtrac ZetaView PMX 100 system, the range distribution of the size was broadly scattered and reached from 90 to 800 nm. Previous studies also revealed that MVs shed from tumor cells appear to be rather heterogeneous in their sizes [4]. However, the procedure for generating the material for electron microscopy may be the reason for the significant change from the raw material to isolated MVs. Because the Microtrac ZetaView PMX 100 measures the MVs sizes from supernatants or MVs isolates without prior processing it appeared to be the superior system for size measurements of small nanoparticles.

Having established a reliable method for the isolation of pure tumor cell derived MVs we addressed the question of their functional impact. First we analyzed if CEACAM1 is able to transduce signals into the MVs as it has been shown to do so in various cell types [14]. Tyrosine-phosphorylation of the cytomplasmic tail of CEACAM1-L was described to be the initial step of the signaling processes. Our results revealed that CEACAM1 present on MVs did not become tyrosine phosphorylated despite treatment with strong stimuli such as peroxide and the plasma membrane permeable constitutive phosphatase inhibitor pervanadate. Interestingly, we could not induce tyrosine phosphorylation of any of the proteins present in the MVs by our treatments. We concluded that the molecular machinery necessary for signaling via tyrosine phosphorylation is either not functioning or absent in MVs. Therefore we postulate that CEACAM1 and other CEACAMs present on MVs function solely as ligand. Our finding that CEACAM1 positive MVs induced strong tyrosine phosphorylation of CEACAM1 in epithelial cells supported this hypothesis. To a lesser extent, also CEACAM1 negative MVs triggered CEACAM1 phosphorylation in epithelial cells. Although the signaling machinery regulating this tyrosine phosphorylation needs to be discovered, it clearly shows the co-stimulatory potential of CEACAM1. Thus CEACAMs on MVs mediate the homophilic/heterophilic interaction with transmembrane anchored CEACAM1 expressed on various cell types, consequently inducing signal transduction and cellular processes.

Baring in mind that MVs interact specifically with cells they recognize via binding to specific, membrane-bound receptors and that both MVs and CEACAMs can be involved in tumor immune suppression we focused on analyzing the putative MVs effect on T-lymphocyte stimulation. We combined the well-established anti-CD3 and anti-CD3/CD28 mAb triggered T-cell stimulation with isolated tumor cell line-derived CEACAM-positive MVs or CHO/CHO-CEACAM1 derived MVs. Our results show a significant increase of T-cell proliferation. Thus, CEACAM1 positive MVs showed a stronger effect than CEACAM1-negative ones and the CEACAM1 binding mAb18/20 significantly decreased the co-stimulatory effect of CEACAM1 bearing MVs. At first this result was unexpected because previous reports described the release of immunosuppressive MVs/exosomes by tumor cells [13]. This has been supported by studies showing expression of FasL, TRAIL and TGF- Beta on tumor derived-exosomes [44]. In that context one should note that most of the previous studies focused on exosomes and not on MVs. Moreover, a negative effect on T-cell stimulation would have been in line with reports stating that CEACAM1 is also an inhibitor of T-cell proliferation [14]. However, more recent reports showed that tumor derived MVs are able to increase stimulated T-cell proliferation [45] and also CEACAM1 can act as intensifying co-receptor of the T-cell receptor [22]. It is likely that the complex composition of different components is responsible for the different functional outcomes in the respective cell types. Further studies analyzing the proteome as well as the RNA load might give a better insight into the functional potential of different MVs. However, because MVs interact specifically with cells they recognize by binding to specific receptors, the CEACAM expression on MVs is clearly a mechanism of interaction between MVs and CEACAM expressing cell types.

The release of CEACAM positive MVs from tumor cells could also be a mechanism by which cells lower the amount of these surface proteins. It is well documented that malignant degeneration of epithelial cells lead to a decreased expression of CEACAM1 [35] and perhaps budding off in MVs is the mechanism of this finding. Tumor cell released MVs may also explain the increased levels of different CEACAMs in serum, plasma and urine of patients suffering tumors and other diseases. Therefore it might be of diagnostic interest to distinguish whether CEACAMs are present in a non-membrane bound soluble form or as protein anchored to the membrane of MVs and exosomes in the different body fluids.

## Materials and Methods

### Cell culture and antibodies

The human colon cancer cell line HT29 was obtained from DSMZ Braunschweig, Germany. The human colon cancer cell line T102/3 was kindly provided by R. Kammerer (FLI, Greifswald, Germany) [46]. The murine cell line (bEnd.3) derived from mouse cortex endothelium was purchased from ATCC (USA). The CHO-huCEACAM1 cell line was generated by stable transfection of CHO-cells with the pcDNA3.1-human CEACAM1-4L vector, subsequent G418 selection and subcloning. HT29, T102/3, CHO, CHO-CEACAM1 and bEnd3 were maintained in Dulbecco’s modified Eagle’s medium (Gibco) supplemented with 10% (v/v) heat-inactivated FCS (PAA), 2 mM L-glutamine (Gibco), 100 µg/ml penicillin (Gibco), and 100 µg/ml streptomycin (Gibco) in a humidified 5% CO_2_ at 37°C atmosphere. The human microvascular endothelia cell line AS-M.5 [47] was cultured in ECG medium (PromoCell).

The different mAbs specific for CEACAM1 (B3-17, C5-1X), CEACAM5 (5C8C4), CEACAM6 (1H7-4B) and CEACAM1/3/5 (18/20) were generated by B.B. Singer (Anatomy, University Hospital Essen, Germany). The mouse mAb anti mouse CEACAM1 was kindly provided by K. Holmes (University of Colorado, USA). The phospho-tyrosine mAb 4G10 was purchased from MerckMillipore (Schwalbach, Germany), the CD29 (clone MEM101-A), CD49b (clone 10-G11) CD3 (clone UCHT-1) and CD28 (clone 15E8) were from Immunotools (Friesoythe, Germany).

### Isolation of MVs

The different adherent growing epithelial, endothelial, CHO and CHO-CEACAM1 cell lines were cultured in 20 ml medium until they reached tight confluence. Then the cells were washed twice with PBS and cultured under starving condition in DMEM medium lacking FCS to provoke stress related release of MVs. The cell culture supernatants were collected after 72 hours and centrifuged at 1 500 g for 20 minutes to remove cells and bigger cells debris. Thereafter supernatants were filtered through a 0.8 µm filter (Pall Life Sciences, Dreieich, Germany) to remove larger cell organelles, vesicles and apoptotic fragments and centrifuged at 41 000 g for 1 hour at 4°C. The supernatant was discarded and the MVs in the pellets were resuspended in 30 ml PBS and centrifuged at 41 000 g for 1 hour at 4°C. The MVs pellets were resuspended in 100 µl 4°C PBS and stored at 4°C until further use. The protein concentration of the MVs isolates was determined by the Lowry protein assay (Bio-Rad, Munich, Germany) according to manufacturer’s instructions.

### Nano particle tracking analysis (NTA)

NTA was performed by using the ZetaView (Particle Metrix, Meerbusch, Germany). The instrument tracks the Brownian motion of laser beam illuminated particles over time and subsequently calculate particle sizes and concentrations. Comparable to exosome-enriched fractions, 1:500 or 1:1000 H_2_O diluted MV probes were measured in duplicates [48].

### Electron microscopy

Cells were cultured on Thermanox® wafers for 3 days in DMEM medium with and without FCS. Medium was withdrawn by careful suction and replaced by 2 ml of a solution of 2.5% glutaraldehyde in 0.1 Mol cacodylate buffer (pH 7.4; room temperature). This was followed 90 min. later by washing in cacodylate buffer (3x 20 min). Osmification with 1% osmiumtetroxide in cacodylate buffer for 60 min was followed by washing in cacodylate buffer (3x 20 min). Then aqueous ethanol solutions were applied (30% -45 min, 50% -45 min.) and 70%. The latter solution also contained 1% of uranyl acetate and was incubated for 60 min. After this 80%, 90%, and 96% ethanol (45 min each) and pure ethanol were applied (3x 10 min each) followed by EPON^®^ solutions in ethanol with increasing EPON^®^ concentration (ethanol: EPON^®^ = 3:1, 1:1, 1:3; 60 min each) and finally pure EPON^®^ overnight at room temperature. Embedding was performed in flat embedding molds whereby Thermanox^®^ wafers were put upside-down on the molds avoiding air bubbles. A heated storage (60°C, 2 days) was used for polymerization. Then Thermanox^®^ wafers were removed using a crimper on a heating plate at 90°C and peeled off from the underlaying Epon. After trimming solid EPON^®^ blocs were cut on a Reichert-Jung Ultracut^®^ ultramicrotome set to a thickness of 60 nm. Sections were then mounted on 200 Mesh hexagonal copper grids and treated with 1% uranyl acetate solution for 4 min. In several, but not all cases this was followed by 3 min of lead citrate (0.4%) for contrast enhancement. A Zeiss transmission electron microscope (EM 902A) was used for final investigation at 80 KV at magnifications from 3 000 to 140 000 x. Digital image acquisition was performed by a MegaViewII slow-scan-CCD camera connected to a PC running ITEM ^®^5.0 software (Soft-imaging-systems, Münster, Germany). Images were stored as uncompressed TIFF files in 16 bits of gray and further processed using Adobe®Photoshop®CS5.

### Western blotting

A fraction of the harvested cells and their corresponding MVs were lysed on ice for 30 min in RIPA based lysis buffer [50 mM Tris-HCl, pH 7.5, 150 mM NaCl, 1% Triton X-100, 0.5% sodium deoxycholate) supplemented with protease inhibitor cocktail set III (Calbiochem) and PhosSTOP phosphatase inhibitor cocktail (Roche). Lysates were centrifuged at 10 000 g at 4°C for 15 min and 50 µg of total protein was subjected to Tricine-PAGE, blotted to nitrocellulose membrane (Applichem, Germany) and reacted with respective primary anti-CEACAM1, anti-CEACAM5, anti-CEACAM6 and anti P-Tyr antibody followed by HRP-coupled secondary goat anti mouse antibody (Dianova, Germany). Anti-β-actin antibody was used to confirm equal loading. Then Western blots were reacted with luminol solution and the chemiluminescence reaction was visualized by the LAS3000 Image analyzer (Fuji, Japan).

### Immunoprecipitation

A fraction of the indicated cells, their corresponding MVs and HT29 cells cultured for 15 min with and without 50 µM pervanadate (Pierce, Germany) as well as CHO- and CHO-CEACAM1 derived MVs were lysed in RIPA based lysis buffer supplemented with protease inhibitor cocktail set III (Calbiochem) and PhosSTOP phosphatase inhibitor cocktail (Roche). Cell and MVs lysates were centrifuged at 10 000 g at 4°C for 15 min. For preclearance, samples were incubated for 1 hour at 4°C with 30 µl protein G sepharose 4 Fast Flow beads (GE Healthcare, USA). Then, the samples were centrifuged for 1 minute at 800 g and the supernatant transferred into fresh tubes. 5 µg of the CEACAM1 binding mAb 18/20 was added to the lysates and incubated for 2 hours at 4°C under permanent rotation followed by incubation with 30 µl protein G sepharose at 4°C overnight. The antigen–antibody-protein G bead complexes were pelleted by centrifugation at 400 g for 30 sec and washed three times with PBS. Thereafter, 60 µl Laemli buffer containing 75 mM β-mercaptoethanol was added to the dry pellet and samples were boiled at 98°C for 10 min. Subsequently, the samples were centrifuged at 2300 g for 1 min and equal volumes of the probes were applied to Tricine-PAGE and transferred onto nitrocellulose membrane as described above.

### Flow cytometry

Cells (5×10^5^) and MVs were stained with mAbs (20 µg/ml) anti-human CEACAM1 (B3-17) and anti-mouse CEACAM1 (CC1) diluted in 3% FCS/PBS for 1 h on ice, washed with ice-cold PBS, and incubated with FITC conjugated anti-mouse F(ab')2 (Dianova, Germany). Background fluorescence was determined using isotype-matched Ig mAb. The stained cell samples were examined in a FACScalibur flow cytometer (BD Biosciences, San Diego, CA) and the data were analyzed utilizing the CellQuest software. Where applicable, dead cells, identified by PI staining, were excluded from the determination. In some cases the content of unstained MVs present in the different cell culture supernatants was determined by measuring the samples for one minute (counts per minute).

### Tyrosine phosphorylation

A fraction of confluent HT29 cells and corresponding MVs were treated for 15 min. at 37°C with 50 µM freshly prepared pervanadate (Pierce, Germany). Another fraction was incubated with 0.03% H_2_O_2_ or left untreated. In addition, confluent HT29 cells were treated with CHO or CHO-CEACAM1 derived MVs, respectively. Thereafter samples were immediately harvested by centrifugation and lysed in RIPA buffer supplemented with protease inhibitor cocktail set III (Calbiochem) and PhosSTOP phosphatase inhibitor cocktail (Roche) for 30 min on ice. The insoluble material was removed by centrifugation at 18 620 g at 4°C for 10 minutes. Subsequently the lysates were either used for the CEACAM1 immunoprecipitation or directly applied for Western blot analyzes.

### Peripheral blood mononuclear cells (PBMC) isolation

Heparinized venous blood was obtained from healthy volunteers in accordance with a protocol approved by the Ethical Review Committee of the University Duisburg-Essen. The participants provided their written informed consent to participate in this study. PBMC were isolated using Ficoll-Hypaque density-gradient centrifugation followed by hypotonic lysis of contaminating erythrocytes if required. The purity of the isolated PBMCs were 90–96% as determined with the FACSCalibur flow cytometer utilizing the CellQuest software (BD Biosciences) with a viability of >97% as measured by trypan blue exclusion. PBMC were resuspended in RPMI (GIBCO) supplemented with 10% (v/v) heat inactivated fetal calf serum (FCS) and 2 mM L-glutamine, 100 IU/ml penicillin, 100 mg/ml streptomycin, and kept on ice until further use.

### PBMC proliferation assays

The proliferation effect of MVs was studied using a BrdU Proliferation Kit (Roche) according to the manufacturers’ protocol. Briefly, 160 000 monocyte-depleted PBMCs/well of a 96 well flat bottom plate were cultured for 3 days with 0.5 µg/ml anti-CD3 and 1 µg/ml anti CD28 in the presence or absence of MVs as described in the figure legends. PBMCs alone or only with MVs served as controls. During the last 18 h BrdU reagent was added into the wells. Then PBMCs were pelleted down, fixed, washed, blocked and incubated with HRP-coupled BrdU detecting antibody. Finally, cells were washed and developed with TMB Xtra-substrate (Kementec Diagnostics). The reaction was stopped with 1 M H_2_SO_4_ and the OD was measured in a Sunrise-ELISA reader (Tecan, Crailsheim, Germany). All measurements were determined in triplicates.

The CFSE approach (CellTrace™ CFSE Cell Proliferation Kit, Molecular Probes™, USA) served as further method for the analysis of cell proliferation. Therefore freshly isolated PBMCs (10^6^ cells/ml) were incubated with 2 µM CFSE for 15 min at 37°C. The cell labeling was quenched by adding five volumes of ice-cold RPMI (10% FCS) for 5 min. Then the cells were washed twice in ice-cold RPMI 1640 (Gibco). According to previous pilot experiments revealing the cell culture condition in which PBMC barely react to CD3 and CD3/CD28 stimulation, 100.000 cells/2.2 ml were cultured in the presence or absence of anti CD3 (0.5 µg/ml, clone UCHT-1) and anti CD3 plus CD28 (1 µg/ml CD28) with or without MV isolated from indicated cell types in 24-well plates (Nunc, Denmark) at 37°C, humid atmosphere, 5% CO_2_ in RPMI supplemented with antibiotics and 10% autologous plasma. In some cases CEACAM1 binding mAb18/20 (50 µg/ml) or isotype matched control IgG (50 µg/ml) was applied to the samples. Untreated PBMCs served as negative control for cell reactivity. After 4 days of cultivation, the cells were collected and analyzed utilizing the Accuri C6 system (Becton Dickinson), 50.000 events per sample were acquired and the viable lymphocytes were selected according to their forward (FSC) and side scatter (SSC) characteristics. Then, the CFSE fluorescence profile of the PBMCs was displayed as histogram and the fraction of proliferating PBMCs was determined.

### Statistical Analysis

Where applicable, data are presented as the mean ± SD. Statistical significance was determined utilizing the Student’s t-test. Differences were considered statistically significant at a P value of <0.05.
